# Factors associated with social support for family members who care for stroke survivors

**DOI:** 10.3934/publichealth.2022011

**Published:** 2021-12-13

**Authors:** Anna Kavga, Ioannis Kalemikerakis, Theocharis Konstantinidis, Ioanna Tsatsou, Petros Galanis, Eugenia Karathanasi, Ourania Govina

**Affiliations:** 1 Department of Nursing, University of West Attica, Athens, Greece; 2 Department of Nursing, Hellenic Mediterranean University, Heraklion, Crete, Greece; 3 Oncology-Hematology Department, Hellenic Airforce General Hospital, Athens, Greece; 4 Department of Nursing, National and Kapodistrian University, Athens, Greece; 5 Department of Nursing, Dafni, Attica Psychiatric Hospital, Athens, Greece

**Keywords:** stroke, family, caregiver, social support

## Abstract

**Introduction:**

Vascular strokes are a primary cause of long-term disability for adults, with many social consequences for the patient, the family and healthcare systems worldwide.

**Aim:**

To investigate the relation between patients' and caregivers' characteristics, as well as burden and depression, and the social support received by carers for stroke victims in Greece.

**Method:**

Patients and caregivers were recruited from community settings in the Attica region of Greece, using purposive sampling. They completed a set of questionnaires during face-to-face interviews. Correlational and multiple regression analyses were performed to identify factors associated with caregivers' perceptions of social support.

**Results:**

In total, 109 dyads of patients and their respective caregivers were recruited. The patients' mean age was 69.3 years, while caregivers' mean age was 58.0 years; 51.4% of patients were males, whereas 67.9% of the caregivers were females. The majority of both patients and caregivers were married, with an annual family income less than €10,000. The level of perceived social support was significantly associated with patients' or caregivers' annual family income, caregivers' working status and the daily caring hours (p < 0.01). Greater perceived support was significantly associated with a lower care burden BCOS (r = 0.29, p < 0.01) and female gender (p = 0.023), but not with the patient's functional level nor with depression (p > 0.05).

## 1. Introduction

Vascular stroke is a leading cause of long-term and persistent disability in adults, thus making stroke victims the most frequent users of healthcare services. In Greece, limited data exists regarding the epidemiology of strokes. In a prospective population-based study of acute first ever stroke incidences, during a 1-year period, 36.8% patients died [1]. Survivors of stroke experience significant functional, cognitive and emotional consequences that may severely affect their daily lives [2],[3].

Caring for a stroke survivor demands changing the lives of the whole family involved in the provision of care. Inevitably, the family becomes the informal caregiver, playing a pivotal role in supporting survivors as they transition through the healthcare system and re-establish themselves in the community setting. However, this often comes at a substantial personal cost [4]. Caregivers may neglect their own health and can experience negative psychological outcomes, such as depression and anxiety [5],[6], as well as a decrease in participation in activities and everyday life roles (e.g. employment, hobbies, social interactions) that have been consistently associated with well-being [4],[7]. The extensive and demanding care results in negative effects that are described as caregiver burden, tension and stress. The burden of care refers to the negative emotions and tension experienced by the caregiver as a result of caring for patients with stroke or other chronic condition [8]. Caregiver burden also seems to be correlated with a reduction in the social support caregivers receive [9] and may prevent their participation in meaningful activities, with a negative effect on life satisfaction [9],[10]. Social support is a broad term that includes all the sources from which individuals may receive support in their social context [11]. These include family (e.g., being married), professional status (e.g., being employed with an adequate income versus being unemployed), and other personal relationships (e.g., friends and neighbors). A caregiver's perceptions of social support may have an impact on the post-stroke patient's recovery process.

Social support can be classified into structural or functional aspects. Structural measures refer to the existence of social relationships and have a linear relation with people's quality of life. Functional measures, on the other hand, refer to resources and services provided by people within an individual's social network, and are typically what people think of when considering social support. In addition, there are further categories of functional measures, such as emotional, instrumental and informational support [12].

The concept of social support emerged in studies of the 1970s. It can be defined as the perceived availability, or actual provision of a relationship, information or assistance, that empowers a person to manage their day-to-day life effectively in the presence or absence of a crisis [12]. Changes in family structures, like the appearance of smaller or dispersed families [13], lead to a reduction in the number of people available to support the many and complex needs of stroke survivors. Inevitably, therefore, caring roles are often undertaken by close relatives, especially spouses, who in many cases must deal with medical conditions of their own or the effects of aging [14], and in most cases are without adequate or consistent social support [4].

Social support has been found to impact people's health, well-being and their ability to adjust to crises or trauma [4],[15]. Stroke research studies began in the 1990s to include social support as a predictor variable, and it has been found to enhance the outcomes associated with stroke recovery. Although few related studies exist, they have investigated different aspects of social support, such as the types, timing and structure of support [15].

The aim of this study was to investigate the relation between patients' and caregivers' characteristics and the social support received by carers for stroke victims in Greece. In addition, we examined the association between social support, burden and depression in family caregivers of stroke survivors.

## 2. Methodology

### 2.1. Study design

A cross-sectional study was conducted via a convenience sampling of dyads consisting of stroke survivors and their primary family caregivers.

### 2.2. Participants and setting

The population of this study consisted of 109 stroke survivors with functional problems and their 109 primary caregivers, who were members of their family, living in the Attica region of Greece (Athens and suburbs).

The selection criteria for the patients included their inability to perform basic or complex functional activities of daily living, owing to their stroke, and the passage of 4 months from the stroke. In particular, the primary researcher communicated with neurologists and physicians from the private sector, the Hellenic Red Cross Home Care Service and the National Rehabilitation Center and identified the eligible patients. A telephone conversation with the family caregiver followed, in which information about the patient's general condition was requested. At this stage, stroke survivors who were already self-sufficient were excluded from the study. A meeting was then arranged at the patient's home to complete the questionnaires.

For caregivers, the inclusion criteria were to be a family member, to have the main responsibility for patient care and to be co-resident with the care recipient. People who did not speak Greek well and those whose medical records showed pre-existing mental or psychiatric problems were excluded from the study.

The collection of the sample lasted 13 months and was carried out in the community setting during home visits (approximately 1-hour duration).

### 2.3. Ethics

During home visits, the patient and the caregiver were verbally informed about the purpose of the study, confidentiality, anonymity, voluntary participation and the possibility of leaving the study at any time they so wished; they were then asked to sign a formal consent form. Permissions were obtained from the Ethics Committee of the Nursing Department of The National and Kapodistrian University of Athens, the Nursing Department of the Hellenic Red Cross and the Board of Directors of the National Rehabilitation Center (protocol numbers: 133-6/10/2014, 1566-21/7/2014, 452-23/5/2014).

### 2.4. Instrumentation

Patients completed a set of questionnaires that included demographic and clinical characteristics and a functionality rating scale (Barthel Index) [16]. In addition, caregivers provided demographic information, and were also graded according to a caregiving outcome scale (Revised Bakas Caregiving Outcomes Scale—Greek version) [17],[18], a depression measurement scale (Center for Epidemiological Studies-Depression, CES-D) [19] and finally a social support scale (Personal Resource Questionnaire, PRQ 2000) [20].

### 2.5. Barthel Index (BI)

The Barthel Index (BI) was used to assess the patient's functional capacity. The scale focuses on the patient's functional capacity for the basic activities of daily living at home. Middle categories imply that the patient supplies over 50 per cent of the effort (total score: 0–100). The original scale of 10 questions [16] was used. Cronbach's alpha coefficient in the present study was very good (0.95).

### 2.6. Revised Bakas Caregiving Outcomes Scale—Greek version (G-BCOS) (family caregivers)

The revised Greek BCOS is a 15-item questionnaire that assesses caregivers' perceptions of changes in their lives as a result of providing care for the patient [18]. The 15 BCOS items measure changes in the physical health, social functioning and subjective well-being of caregivers on a 7-point scale. The numbers 1, 2 and 3 represent a large, medium or small change for the worse; the number 4 means that there was no change; and the numbers 5, 6 and 7 indicate a small, medium or large change for the better. The possible range of values on the scale ranges from 15 to 105. An overall score of <40 indicates that caregivers believe that the changes in their lives due to care are for the worse. The Cronbach's alpha coefficient for the total Greek BCOS score in the present study was 0.87.

### 2.7. Center for Epidemiological Studies-Depression (CES-D)

The CES-D scale focuses on the depressive symptoms of the non-psychiatric population, such as caregivers of patients with stroke. Its Greek version has satisfactory validity and reliability [21],[22]. It includes 20 questions and six subscales that refer to physical and mental symptoms indicative of depression. The answers are given on a 4-point scale (0 = rare/not at all, up to 3 = most of the time). The overall score ranges from 0 to 60, with the cut-off point separating normal from psychopathological condition at 16 [19]. A higher score indicates a higher level of depression [22]. In the present study, Cronbach's alpha reliability coefficient was also satisfactory (0.84).

### 2.8. Personal Resource Questionnaire (PRQ 2000)

PRQ 2000 [20] is designed to measure perceived social support and includes 15 questions. It is graded on a 7-point Likert scale and shows the degree of the respondent's agreement or disagreement (from 1 = strongly disagree to 7 = strongly agree). The score of the scale ranges from 15 to 105, with the highest score being an indication of a higher level of perceived social support. For the needs of the present study, Cronbach's alpha coefficient was very good (0.84).

**Table 1.** Sample characteristics

	N (%)
*Patients*	
Gender	
Males	56 (51.4)
Females	53 (48.6)
Age, mean (SD)	69.3 (13.7)
Educational level	
Primary school at most	65 (59.7)
Middle/high school or university	44 (40.3)
Married	72 (66.1)
Number of children, mean (SD)	2.2 (1.3)
Family members living in the same house, mean (SD)	2.8 (1.1)
Annual family income	
<€10,000	62 (56.8)
>€10,000	47 (43.2)
Equipment and facilities in the house	39 (35.8)
Diagnosis	
Right hemiplegia	52 (47.7)
Left hemiplegia	57 (52.3)
Months in need of care, median (IQR)	10 (5–36)
*Caregivers*
Gender	
Males	35 (32.1)
Females	74 (67.9)
Age, mean (SD)	58.0 (13.5)
Relation with patient	
Spouse	55 (50.5)
Son/daughter	38 (34.9)
Other	16 (14.7)
Educational level	
Primary school at most	38 (34.9)
Middle/high school or university	71 (65.1)
Married	83 (76.1)
Number of children, mean (SD)	1.7 (1.1)
Working status	
Employed	33 (30.3)
Pensioner	58 (53.2)
Other	18 (16.5)
Annual family income	
< €10,000	59 (54.1)
> €10,000	50 (45.8)
Months taking care of the patient, median (IQR)	8 (5–25)
Daily hours of care, mean (SD)	13.2 (6.4)
Health condition	
Good	44 (40.4)
Moderate	54 (49.5)
Poor	11 (10.1)

Note: SD: standard deviation.

### 2.9. Statistical analysis

Quantitative variables were expressed as mean values [± standard deviation (SD)] or median [interquartile range (IQR)], while qualitative variables were expressed as absolute and relative frequencies. Student's t-tests and analysis of variance (ANOVA) were computed for the comparison of mean values. The Bonferroni correction was used in case of multiple testing in order to control for type I error. Pearson or Spearman correlation coefficients were used to explore the association of two continuous variables. Multiple linear regression analysis was used with dependent variable the PRQ-2000 scale. The regression equation included terms for patients' characteristics, caregivers' characteristics, as well as Barthel Index, CES-D and BCOS scale. Adjusted regression coefficients (β) with standard errors (SE) were computed from the results of the linear regression analyses. All reported p-values are two-tailed. Statistical significance was set at p < 0.05 and analyses were conducted using SPSS statistical software (version 22.0).

## 3. Results

The characteristics of the 109 patients and their caregivers are presented in table 1. The patients' mean age was 69.3 years (SD = 13.7 years) and the caregivers' mean age was 58.0 years (SD = 13.5 years); 51.4% of the patients were males, whereas 67.9% of the caregivers were females. Approximately half of the caregivers (50.5%) were their patient's spouse. The majority of both patients and caregivers were married, with an annual family income less than €10,000. Regarding the type of stroke, 52.3% of the patients were diagnosed with left hemiplegia and 47.7% with right hemiplegia. The caregivers' health condition was moderate in 49.5% and good in 40.4%.

Descriptive statistics of all scales under study are presented in table 2. PRQ 2000ranged from 43 to 102, with mean 77.7 (SD = 13.8).

**Table 2.** Descriptive statistics of study scales

	Minimum	Maximum	Mean	Standard deviation
Greek BCOS	20	86	48.3	13.3
Barthel Index	0	95	44.0	30.6
CES-D	5	47	21.7	10.1
PRQ 2000	43	102	77.7	13.8

The univariate analysis revealed that caregivers' evaluation of social support was significantly higher when patients' or caregivers' annual family income was above €10,000 euro (p < 0.005) (Table 3). Also, social support varied significantly in relation to the caregivers' working status. More specifically, after a Bonferroni correction, social support was found to be significantly greater in cases where the caregiver was employed, compared to when he/she had other working status (p = 0.024). In addition, more hours of daily care were significantly associated with less social support (p = 0.030). PRQ 2000 was not significantly associated with the Barthel Index (r = 0.01, p > 0.05) nor with CES-D (r = −0.12, p > 0.05). However, PRQ 2000 was positively significantly associated with BCOS (r = 0.29, p < 0.01).

**Table 3.** Univariate analysis for PRQ 2000 with patients' and caregivers' characteristics.

	PRQ 2000	
	Mean (SD)	P
*Patients*		
Gender		
Males	77.8 (13.9)	0.896^+^
Females	77.5 (13.8)	
Age, r^1^	0.02	0.836
Educational level		
Primary school at most	77.9 (13.5)	0.806^+^
Middle/high school or university	77.3 (14.2)	
Married		
No	80.5 (10.5)	0.130^+^
Yes	76.2 (15.0)	
Number of children, r^1^	0.08	0.389
Family members living in the same house, r^1^	−0.01	0.912
Annual family income		
<€10,000	74.5 (13.8)	0.005^+^
>€10,000	81.8 (12.8)	
Equipment and facilities in the house		
Yes	76.9 (15.5)	0.674^+^
No	78.1 (12.8)	
Diagnosis		
Right hemiplegia	80.1 (10.7)	0.217^+^
Left hemiplegia	75.3 (15.1)	
Months in need for care, r^2^	0.06	0.534
*Caregivers*		
Gender		
Males	74.3 (15.2)	0.080^+^
Females	79.3 (12.8)	
Age, r^1^	−0.11	0.237
Relation with patient		
Spouse	75.9 (15.0)	0.393^++^
Son/daughter	79.3 (13.0)	
Other	80.0 (10.6)	
Educational level		
Primary school at most	76.1 (15.5)	0.396^+^
Middle/high school or university	78.5 (12.8)	
Married		
No	78.8 (14.2)	0.643+
Yes	77.3 (13.7)	
Number of children, r^1^	0.13	0.183
Working status		
Employed	82.6 (10.9)	0.020^++^
Pensioner	76.6 (14.1)	
Other	72.1 (15.2)	
Annual family income		
<€10,000	75.3 (14.4)	0.050^+^
>€10,000	80.5 (12.5)	
Health condition		
Poor/moderate	76.9 (13.6)	476^+^
Good	78.8 (14.0)	
Months taking care of the patient, r^2^	−0.01	0.931
Daily hours of care, r^1^	−0.21	0.030

Note: ^+^Student's t-test; ^++^ANOVA; ^1^Pearson's correlation coefficient; ^2^Spearman'scorrelation coefficient; SD: standard deviation.

Then, in order to determine which factors affected the variable of social support, we conducted multiple regression analysis with PRQ 2000 as dependent variable and the following variables were entered in the model: BCOS, CES-D, BI, caregiver's gender, age and educational level, the relationship with the patient, caregiver's working status, daily hours of care provided and annual family income.

When multiple regression analysis was conducted it was found that in cases where the caregiver was a woman PRQ 2000 score was significantly higher (greater social support). On the contrary in cases where the caregiver had “other working status”, PRQ 2000 score was significantly lower compared to cases where the caregiver was employed. Furthermore, higher BCOS score (lower burden) was significantly associated with greater social support (Table 4).

**Table 4.** Regression analysis results with PRQ 2000 as dependent variable and caregivers' characteristics, BI, CES-D, and BCOS as independent variables.

	β+	SE++	P
Caregiver's Gender			
Males (reference)			
Females	7.17	3.11	0.023
Caregiver's Age	0.11	0.16	0.487
Caregiver's Educational level			
Primary school at most (reference)			
Middle/High school or University	1.98	3.05	0.517
Relation with patient			
Son/Daughter (reference)			
Spouse	−3.43	3.62	0.346
Other	−1.07	4.69	0.820
Caregiver's Working status			
Employed (reference)			
Pensioner	−3.68	4.48	0.414
Other	−8.47	4.33	0.049
Daily hours of care	−0.14	0.26	0.589
Annual Family Income			
< 10,000€ (reference)			
> 10,000€	2.27	2.90	0.436
Greek BCOS	0.31	0.11	0.007
CES-D	0.07	0.15	0.662
Barthel Index	−0.04	0.05	0.396

Note: ^+^regression coefficient; ^++^Standard Error.

## 4. Discussion

This study evaluated the factors that were associated with the social support received by caregivers of stroke patients. A patient's higher level of income affected caregivers' perceived social support, reflected in a higher PRQ score. Females, those caregivers who were currently employed and had a high income also reported greater social support, whereas support was lower in those involved with many hours of daily care. Greater perceived support was significantly associated with a lower care burden BCOS (r = 0.29, p < 0.01).

Thus, a higher income for both patients and carers appears to have a positive effect on the carers' perception of social support. It is possible that relative financial comfort enables the caregiver to obtain some form of assistance at an appropriate cost, leaving time for social interactions. Income level is often associated with health status and social connectedness: the greater the income, the greater the potential for access to health care and social interactions [23]. Feelings of loneliness and social isolation as a result of caring responsibilities seem to be mitigated by income [24], whereas special care requirements may negatively affect financial status [25]. It is therefore possible that the health-related effects of care on caregivers' lives are positively influenced by factors such as income and social support [26]. Low-income carers are considered to belong to a vulnerable group [27]. Factors such as loss of income, cost savings and working hours can lead to a shift away from most social and daily activities, resulting in greater caregiver distress [28]. In Greece, there is no legislation regarding the provision of care by caregivers. There are only isolated efforts by voluntary organizations to support family caregivers in the community and municipal programs for “help at home”. In addition, in some cases a very small allowance is granted to chronically ill people for their care needs, depending on their income and degree of disability. An increase in income could help reduce the burden on carers, as it would allow them to secure some paid respite care for a few hours and reduce their financial distress.

Evidence regarding the impact of employment on the care experience is conflicting. In the present study, caregivers in employment experienced higher social support. Employment status could act as a protective factor against the accumulation of distress [29]. Communication and interpersonal relationships play an important role in caregiver support, as shown in the present study, where working caregivers felt they received significantly more social support compared to those who were retired. In contrast, many hours of daily care were found to negatively affect social support. Obviously, caregivers with only limited time available were not able to develop and maintain a social network around themselves. Supporting caregivers in fulfilling social roles and performing important life activities has a beneficial effect on maintaining good health and well-being and on their ability to continue to provide long-term care to the patient [10]. Overall, in the literature, employment status seems to be associated with caregivers' burden. The coexistence of positive and negative assessments is common in various studies of caregivers' burden [30].

Patients, too, can benefit from social support, given the direct relationship between family caregivers and patients' quality of life [31]. High levels of functional measures of social support (family support, instrumental, emotional) are associated with a progressive improvement in functional status, mainly in severely impaired patients. Overall, patients receiving a high level of support have significantly better social status [32], which affects caregivers' everyday lives.

We also found a significant positive correlation between social support (greater PRQ 2000) and caregivers' burden score, with a higher BCOS score being indicative of a smaller burden. Social support seems to be beneficial for caregivers' health [33]. Chung et al. (2020) [34] assessed 102 caregivers in South Korea at two points: post-discharge and one year after the patient's stroke. Persistent depressive symptoms were linked to the highest level of burden, and the lowest levels of family function and perceived availability of social support at both assessment times. Moreover, a low level of municipal social service support was associated with a significantly greater caregiver burden [35], while high social support helped to limit the burden for caregivers [36]. In contrast to our results, those authors found no variables apart from caregiver's burden and mental health that were associated with social support [34].

Notably, in the majority of reports in the literature the primary caregiver was a woman (spouse/ partner/daughter). Women are the typical primary caregivers in the family setting. They appear to see it as a moral obligation and they feel resigned to being assigned the role of caregiver, even if they do not recognize this themselves. As a result, female caregivers whose physical and psychological health is affected have higher levels of care burden and lower levels of social support [34]. Notably, in our study women experienced higher levels of social support than men caregivers.

A survey in seven European countries found that social support varies from country to country and depends on many factors [37]. In Greece, social support is observed to a greater extent than in other countries. This positive relationship with perceived social support may be interpreted in terms of the greater family bonds that exist in our country.

The limitations of this study included the relatively small samples of caregivers and patients, making it difficult to generalize the results to all caregivers and patients affected by stroke in our country. In addition, we acknowledge that our convenience sample may not be representative of the total population of family caregivers of stroke survivors in Greece. Although a large number of potential factors that might affect the perception of social support were investigated in this study, other factors such as the quality of the relationships between members of the household, or the level of communication with the patient him- or herself, could have influenced the need for support and should be investigated in future studies. Finally, longitudinal studies are required to establish how caregiving burden is affected by health changes over time.

## 5. Conclusions

The present study investigated the effects of patients' and caregivers' characteristics on the social support of the family caring for patients with stroke. Higher levels of social support were associated with caregivers' and patients' income, employment status, and the female gender while lower support was related with daily hours of care. A low level of social support was also associated with a higher caregiver burden.

These findings are of major importance, since the present study is one of the few conducted in the Greek population and may increase nurses' awareness of caregivers' need for social support during the care of a person affected by a chronic and dependent condition. Long-term care of stroke patients should include the identification and assessment of vulnerable caregivers in need of support, so that appropriate interventions can be implemented. Given that there is a direct relationship between patients' condition and family caregivers' wellbeing, nurses should pay more attention to family-centered health care. The absence of caregiver support programs increases the use of healthcare services [38]. Therefore, evaluating the role of the caregiver should be a nursing priority to alleviate the stress of caring while mobilizing the appropriate support network through Community Health Centers. Our public healthcare system should try to ensure optimal community and home care in order to support informal caregivers. Funding could be found from volunteer or scientific organizations, or even national and European funds.

Future research could focus on prospective studies and on monitoring the same dyads (caregiver/patient) at different times during care. In addition, this study could act as a trigger for more research about informal care and policy making and its impact on the health of caregivers, in order to highlight the multiple needs of caregivers and at the same time to stress the need and importance of community and family nursing.
